# Histopathological comparison of bone healing effects of endonasal and percutaneous lateral osteotomy methods in rabbit rhinoplasty model^☆^

**DOI:** 10.1016/j.bjorl.2017.06.008

**Published:** 2017-07-17

**Authors:** Şahin Öğreden, Sedat Rüzgar, Hasan Deniz Tansuker, Ümit Taşkın, Yalçın Alimoğlu, Salih Aydın, Mehmet Faruk Oktay, Uğur İzol

**Affiliations:** a University of Health Sciences, Bagcilar Training and Research Hospital, Department of Otolaryngology, Istanbul, Turkey; b Haseki Training and Research Hospital, Department of Otolaryngology, Istanbul, Turkey; c University of Health Sciences, Bagcilar Training and Research Hospital, Department of Pathology, Istanbul, Turkey

**Keywords:** Endonasal osteotomy, Percutaneous osteotomy, Rhinoplasty, Animal model, Osteotomia endonasal, Osteotomia percutânea, Rinoplastia, Modelo animal

## Abstract

**Introduction:**

Lateral osteotomy is mainly performed either endonasally or percutaneously in rhinoplasty which is a frequently performed operation for the correction of nasal deformities. Both techniques have both advantages and disadvantages relative to each other.

**Objective:**

The aim of this study was to compare the histopathological effects of endonasal and percutaneous osteotomy techniques performed in rhinoplasty on bone healing and nasal stability in an experimental animal model.

**Methods:**

Eight one year-old New Zealand white rabbits were included. Xylazine hydrocloride and intramuscular ketamine anesthesia were administered to the rabbits. Endonasal osteotomy (8 bones) was performed in Group 1 (*n* = 4), and percutaneous osteotomy (8 bones) in Group 2 (*n* = 4). One month later the rabbits were sacrificed. Bone healing of the rabbits was staged according to the bone healing score of Huddleston et al. In both groups, nasal bone integrity was assessed subjectively.

**Results:**

In the percutaneous osteotomy group, Grade 1 bone healing was observed in two samples (25%), Grade 2 bone healing in two samples (25%), Grade 3 bone healing in four samples (50%). In the endonasal osteotomy group, Grade 1 bone healing was observed in 6 samples (75%) and Grade 2 bone healing was observed in 2 samples (25%). In the percutaneous group, fibrous tissue was observed in 2, predominantly fibrous tissue and a lesser amount of cartilage was observed in 2 and an equal amount of fibrous tissue and cartilage was observed in 4 samples. In the endonasal group, fibrous tissue was observed in 6 samples, and predominantly fibrous tissue with a lesser amount of cartilage was observed in 2 samples. In both groups, when manual force was applied to the nasal bones, subjectively the same resistance was observed.

**Conclusion:**

Percutaneous lateral osteotomy technique was found to result in less bone and periost trauma and better bone healing compared to the endonasal osteotomy technique.

## Resumo

**Introdução:**

Nas rinoplastias, a osteotomia lateral é realizada principalmente por via endonasal ou percutânea para correção de deformidades nasais. Ambas as técnicas apresentam vantagens e desvantagens.

**Objetivo:**

Comparar os efeitos histopatológicos sobre a cicatrização óssea e estabilidade nasal entre as técnicas de osteotomia endonasal e percutânea em rinoplastia em um modelo animal experimental.

**Método:**

Foram incluídos oito coelhos brancos da Nova Zelândia de um ano de idade. Hidrocloreto de xilazina e cetamina intramuscular foram administrados aos coelhos como agentes anestésicos. Osteotomia endonasal (8 ossos) foi realizada no Grupo 1 (n = 4) e osteotomia percutânea (8 ossos) no Grupo 2 (n = 4). Um mês depois, os coelhos foram sacrificados. A cicatrização óssea dos coelhos foi avaliada de acordo com o escore de cicatrização óssea de Huddleston et al. Em ambos os grupos, a integridade do osso nasal foi avaliada subjetivamente.

**Resultados:**

No grupo da osteotomia percutânea, observou-se cicatrização óssea de grau 1 em duas amostras (25%), cicatrização óssea de grau 2 em duas amostras (25%), e cicatrização óssea de grau 3 em quatro amostras (50%). No grupo da osteotomia endonasal, observou-se cicatrização óssea de grau 1 em 6 amostras (75%) e a cicatrização óssea de grau 2 foi observada em 2 amostras (25%). No grupo percutâneo, o tecido fibroso foi observado em 2 amostras, enquanto tecido predominantemente fibroso e uma menor quantidade de cartilagem foi observada em 2 e uma quantidade igual de tecido fibroso e cartilagem foi observada em 4 amostras. No grupo endonasal, observou-se tecido fibroso em 6 amostras e tecido predominantemente fibroso com uma menor quantidade de cartilagem em 2 amostras. Em ambos os grupos, quando força manual foi aplicada aos ossos nasais, a mesma resistência foi observada subjetivamente.

**Conclusão:**

A técnica de osteotomia lateral percutânea resultou em menor traumatismo ósseo e periosteal e melhor cicatrização óssea em comparação com a técnica de osteotomia endonasal.

## Introduction

Rhinoplasty is a frequently performed operation for the correction of nasal deformities. Lateral osteotomies are usually performed at the final stage of esthetic surgery.^1^ Because osteotomies are not performed under direct visual observation but by feeling tactile stimulation, they carry the risk of damaging the mucosa, the supporting tissues and the periosteum.^2^ Ideal osteotomy should be reproducible, predictable and be able to produce definite results with good functional outcomes, and soft tissue damage should be minimal.^3^ Lateral osteotomy may cause excessive damage to the intranasal mucosa and periosteum, increased bleeding, excessively mobilized nose, excessive edema, increased ecchymoses, and excessive narrowing of the nose.^4^ The lateral osteotomy performed after the hump resection corrects the open roof deformities, the curved lateral nasal wall and the broad nasal base.^5^ The lateral osteotomy is mainly performed either endonasally or percutaneously. Both techniques have both advantages and disadvantages relative to each other.

The rabbit nasal bone is thinner and elongated than the human nasal bone. In rabbit facial anatomy, both nasal bones fuses to form roof of nasal cavity and forms the dorsal border of the piriform aperture. Nasal bones articulate with the frontal bone at the posterior. In pup rabbits the frontonasal suture is the active growth zone.^6^

Bones have regeneration and repair capacity after fracture. The fracture healing process involves platelets, inflammatory cells, fibroblasts, endothelial cells, osteoclasts and osteoblasts. The most critical role in this process is attributed to osteoblasts because they are responsible for the synthesis and mineralization of the bone matrix.^7^, ^8^

In this study, we aimed to compare histopathologically the effect of endonasal and percutaneous lateral osteotomy techniques on bone healing and to evaluate nasal stability clinically using a rabbit model.

## Methods

Eight one year-old New Zealand white rabbits were included in this study. The rabbits were divided into 2 groups each containing 4. Group 1 included endonasal osteotomy performed rabbits, and Group 2 included the percutaneous osteotomy performed rabbits. Mucosa, periosteum and the soft tissues were not elevated during the internal osteotomy. In both groups the rabbits were given anesthesia with 5 mg/kg xylazine hydrochloride and 35 mg/kg intramuscular ketamine. Then, bilateral incisions superior to their nares were made, and bone was reached in the rabbits in Group 1. Complete osteotomy was performed using guided osteotomy. In Group 2, the nasal bone was exposed by making a skin incision on the back of the nose. Perforating osteotomies were made in the nasal bone using a 2 mm unguided sharp osteotomy and a greenstick fracture was created. The size of the osteotome for the endonasal technique was 2 mm too. At the end of the operation, the incised skin was sutured and closed. One month later, the rabbits were sacrificed by intracardiac administration of a high dose 120 mg pentobarbital. The nasal bones of the rabbits were resected from the bilateral frontal process of the maxillary bone and separated from the nasal spindle of the frontal bone. The specimens were fixed with 10% buffered formaldehyde. Hematoxylin–eosin was used for staining. Bone healing was staged according to the grading described by Huddleston et al.^9^ The progression of fracture-healing in each specimen was quantified with use of a scale that assigns a grade based on the relative percentages of fibrous tissue, cartilage, woven bone, and mature bone in the callus.^9^ 4 μm sections were taken. Using a microscope (B × 51 Japan), histological grading was performed. The grading was as the following: Grade 1 – Fibrous tissue, Grade 2 – Dominantly fibrous tissue less cartilage, Grade 3 – Equal amount of cartilage and fibrous tissue, Grade 4 – Only cartilage tissue, Grade 5 – Predominantly cartilage and small amount of woven bone, Grade 6 – Equal amount of lateral cartilage and immature bone, Grade 7 – Predominantly immature bone and less cartilage, Grade 8 – Totally immature bone, Grade 9 – Immature bone and lesser amount of mature bone, and Grade 10 – Mature (lamella) bone. In both of the groups, bone strength was assessed subjectively by applying manual force to the nasal bones.

This study was approved by the experimental animals local ethics committee of Bağcılar Training and Research Hospital (2015, n° 2015–07).

## Results

**Figure 1:**
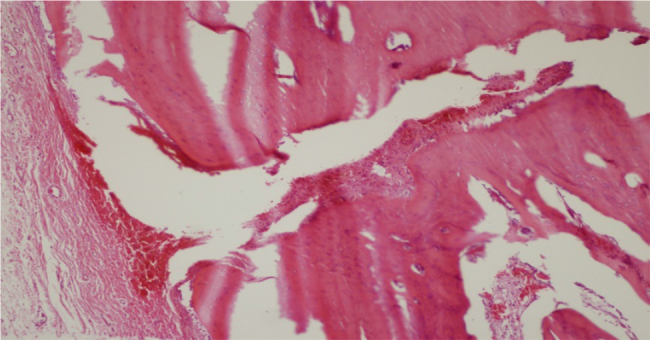
Fibrous tissue and cartilage proliferation in fracture line (H&E ×40).

**Figure 2:**
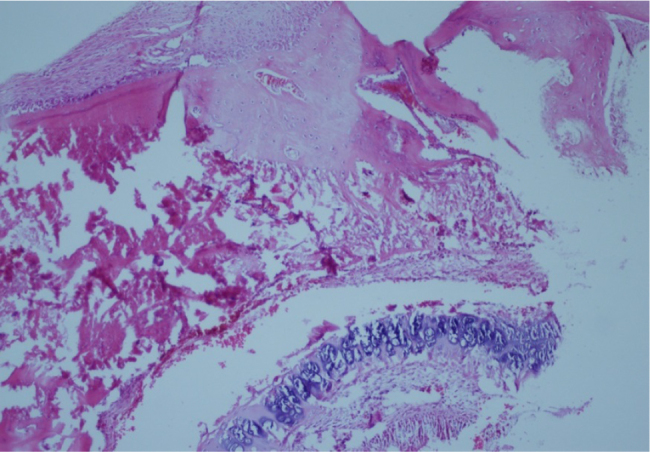
Old bleeding findings in fracture line and fibroblastic proliferation (H&E ×40).

Bone healing was evaluated histopathologically in 16 samples taken from eight rabbits. In the percutaneous lateral osteotomy group, Grade 1 bone healing was observed in 2 samples (25%), Grade 2 bone healing in 2 samples (25%), and Grade 3 bone healing in 4 samples (50%). In the endonasal lateral osteotomy group, Grade 1 bone healing was observed in 6 samples (75%) and Grade 2 bone healing was observed in 2 samples (25%) (Table 1). In the percutaneous group, fibrous tissue was observed in 2, predominantly fibrous tissue and a lesser amount of cartilage was observed in 2 and an equal amount of fibrous tissue and cartilage was observed in 4 samples (Fig. 1). In the endonasal group, fibrous tissue was observed in 6 samples (Fig. 2), and predominantly fibrous tissue with a lesser amount of cartilage was observed in 2 samples (Table 2). In both groups, when manual force was applied to the nasal bones, they had subjectively the same resistance.

**Table 1:** Histological scoring of the effect of endonasal and percutaneous osteotomy techniques on bone healing.

Histological score	Research groups	
	Percutaneous (*n* = 8)	Endonasal (*n* = 8)
1	2 (25%)	6 (75%)
2	2 (25%)	2 (25%)
3	4 (50%)	0
4	0	0
5	0	0
6	0	0
7	0	0
8	0	0
9	0	0
10	0	0

**Table 2:** Histopathological scoring of bone healing.

Histological appearance	Research groups	
	Percutaneous	Endonasal
Fibrous tissue	2 (25%)	6 (75%)
Weighted fibrous tissue and less cartilage	2 (25%)	2 (25%)
Equal amounts of fibrous and cartilaginous tissue	4 (50%)	0 (0%)

## Discussion

Lateral osteotomy is one of the basic procedures in cosmetic nasal surgery. It is usually done to correct open roof deformities after hump resection, to narrow the wide nose or to thin the nasal pyramid. Surgeons generally prefer one of the two primary methods to perform lateral osteotomy; endonasal continuous osteotomy or external perforating osteotomy. The external perforating osteotomy has advantages such as less mucosal and periosteal damage, less nose mobilization due to periosteal stability and more controlled fracture. However, endonasal continuous osteotomy provides more mobilization and more precise narrowing in the nasal pyramid.^4^, ^10^

Rohrich et al. reported that the lateral osteotomy technique caused less bleeding, edema and ecchymoses, and also it provided decreased the risk of nasal instability due to periosteal preservation.^4^, ^10^ In our animal model study, we observed that the technique of percutaneous lateral osteotomy had a positive effect on nasal stability. Rho et al. described internal perforating lateral osteotomy with the wide periosteal elevation and they noted less postoperative ecchymosis, edema, and hemorrhage, and that the patient returned to his or her social life sooner.^11^ Similarly, Hontanilla et al. reported that the patients with external perforating lateral osteotomy where the periosteum was preserved were less likely to have ecchymosis, edema and hemorrhage.^12^ Esteves et al. conducted a study in rats, and examined histological and immunohistochemical properties of bone healing after osteotomy with piezosurgery and classical drilling methods and compared both methods. Although bone healing with piezoosteotomy was superior to conventional osteotomy in the early period, it was reported that it was the same as the conventional method in the late period.^13^ In our study, we have seen that in the rabbit rhinoplasty model, bone healing is superior histologically in the perforated osteotomy technique as compared to endonasal continuous osteotomy.

Inan et al. compared the effect of median and s-shaped sternotomy methods on bone healing and sternal stability in 31 sheep's. They have reported that the stability of the sternum and bone healing were better histologically with s-shaped sternotomy.^14^ In our study with rabbits, we observed histologically superior bone healing with perforating osteotomy technique compared to continuous endonasal technique. However, when manual pressure to the nasal bone was applied, both groups were equally resistant subjectively. Inan and colleagues reported that thorax stability was stronger in the sheep with S-shaped sternotomy. Likewise, Kucukdurmaz et al. reported that a small, non-zigzag-shaped osteotomy was superior to plain osteotomy.^15^ Sinha et al. compared the postoperative effects of endonasal and percutaneous osteotomy techniques during rhinoplasty in 45 patients. Endonasal osteotomy has been reported to cause ecchymosis, edema and hemorrhage more frequently when compared to percutaneous lateral osteotomy.^16^ Gryskiewicz et al. reported that in 50 patients with lateral osteotomy, patients with percutaneous lateral osteotomy had less ecchymosis and edema compared to the endonasal group.^17^ In our study, bone healing was slower and fibrous tissue was predominantly observed in the endonasal osteotomy group compared to the percutaneous osteotomy group. Malhotra et al. studied bone healing histologically and radiologically in 4 sheeps on which they created bone defects of various widths and the same depths in the distal femur and found that bone healing was associated with the defect width.^18^

We concluded that the external perforating osteotomy has advantages in a more controlled fracture and less nose mobilization, which leads to less mucosal and periosteal damage due to periosteal stability. Since this is a histopathological study instead of clinical ones, swelling and bruises were not evaluated postoperatively. The major disadvantage of this study was the low number of the rabbits due to fact that there had been difficulty of ethics committee approval for higher number of rabbits.

## Conclusion

Although the percutaneous lateral osteotomy technique has been shown to be more effective in terms of histological bone healing than the endonasal osteotomy technique, there may be a need for wider series with longer periods of time and correlation with the clinical observation to further clarify this issue.

## Conflicts of interest

The authors declare no conflicts of interest.
